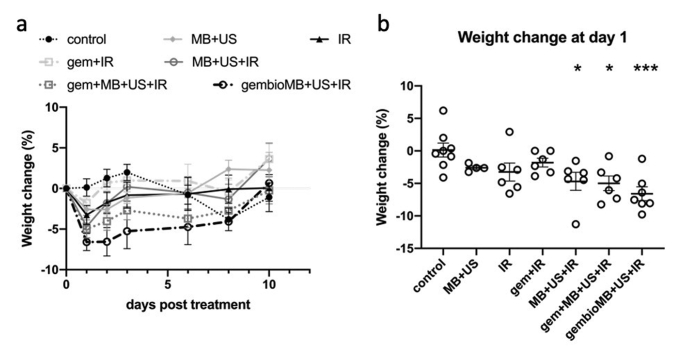# Ultrasound-Mediated Gemcitabine Delivery Reduces the Normal-Tissue Toxicity of Chemoradiation Therapy in a Muscle-Invasive Bladder Cancer Model

**DOI:** 10.1016/j.ijrobp.2020.11.046

**Published:** 2021-04-01

**Authors:** Jia-Ling Ruan, Richard J. Browning, Yesna O. Yildiz, Michael Gray, Luca Bau, Sukanta Kamila, James Thompson, Amy Elliott, Sean Smart, Anthony P. McHale, John F. Callan, Borivoj Vojnovic, Eleanor Stride, Anne E. Kiltie

**Affiliations:** ∗ Department of Oncology, Oxford Institute for Radiation Oncology, University of Oxford, Oxford, United Kingdom; † Institute of Biomedical Engineering, University of Oxford, Oxford, United Kingdom; ‡ Biomedical Sciences Research Institute, University of Ulster, Coleraine, Northern Ireland, United Kingdom

## Abstract

**Purpose:**

Chemoradiation therapy is the standard of care in muscle-invasive bladder cancer (MIBC). Although agents such as gemcitabine can enhance tumor radiosensitivity, their side effects can limit patient eligibility and treatment efficacy. This study investigates ultrasound and microbubbles for targeting gemcitabine delivery to reduce normal-tissue toxicity in a murine orthotopic MIBC model.

**Materials and Methods:**

CD1-nude mice were injected orthotopically with RT112 bladder tumor cells. Conventional chemoradiation involved injecting gemcitabine (10 mg/kg) before 6 Gy targeted irradiation of the bladder area using the Small Animal Radiation Research Platform (SARRP). Ultrasound-mediated gemcitabine delivery (10 mg/kg gemcitabine) involved either coadministration of microbubbles with gemcitabine or conjugating gemcitabine onto microbubbles followed by exposure to ultrasound (1.1 MHz center frequency, 1 MPa peak negative pressure, 1% duty cycle, and 0.5 Hz pulse repetition frequency) before SARRP irradiation. The effect of ultrasound and microbubbles alone was also tested. Tumor volumes were measured by 3D ultrasound imaging. Acute normal-tissue toxicity from 12 Gy to the lower bowel area was assessed using an intestinal crypt assay in mice culled 3.75 days posttreatment.

**Results:**

A significant delay in tumor growth was observed with conventional chemoradiation therapy and both microbubble groups (*P* < .05 compared with the radiation-only group). Transient weight loss was seen in the microbubble groups, which resolved within 10 days posttreatment. A positive correlation was found between weight loss on day 3 posttreatment and tumor growth delay (*P* < .05; R^2^ = 0.76). In contrast with conventional chemoradiation therapy, ultrasound-mediated drug delivery methods did not exacerbate the acute intestinal toxicity using the crypt assay.

**Conclusions:**

Ultrasound and microbubbles offer a promising new approach for improving chemoradiation therapy for muscle-invasive bladder cancer, maintaining a delay in tumor growth but with reduced acute intestinal toxicity compared with conventional chemoradiation therapy.

## Introduction

Bladder cancer is the 10th most common cancer worldwide, with approximately 550,000 new cases each year.^1^ Most are urothelial carcinomas, and approximately a quarter of these are muscle-invasive bladder cancer (MIBC) in which cancer spreads into the detrusor muscle of the bladder wall. Radical cystectomy, the removal of the bladder, has been the standard of care for decades, but the potentially severe outcome on the patient’s quality of life^2^ has made radiation therapy a frequent alternative. Concurrent chemotherapy and radiation therapy has been shown to improve patient survival compared with radiation therapy alone^3^, ^4^, ^5^ and is now also considered as a mainstream treatment option. Gemcitabine-based chemoradiation treatment is widely used, but the toxicity of gemcitabine results in both severe systemic side effects and increased effects of radiation in the healthy bladder tissue surrounding the tumor.^6^, ^7^, ^8^ As many MIBC patients are elderly and unable to tolerate these effects,^8^ improving the delivery of gemcitabine to the tumor site would allow a larger number of patients to receive concurrent gemcitabine-based chemoradiation therapy for MIBC.

Using a combination of microbubbles and ultrasound has been shown to be effective in improving both the penetration of drugs into target tissue and in achieving spatial localization.^9^ Clinically, microbubbles have been used widely as contrast agents for ultrasound imaging. Microbubbles consist of an inert-gas core, typically to 1-10 μm in diameter and stabilized with lipid, polymer, or proteins. On exposure to ultrasound, the microbubbles expand and contract in response to the changing pressure. At low amplitudes, this enhances the echogenicity of the blood, enabling improved image contrast. At higher amplitudes, the microbubble oscillations can lead to increased permeability of both blood vessel endothelia and individual cell membranes and hence increased drug deposition in the exposed tissue.^10^ This effect has been successfully exploited by coinjecting microbubbles with a range of different drugs.^11^, ^12^, ^13^ Improving chemotherapeutic drug uptake by ultrasound-mediated drug delivery has been extensively demonstrated in vitro*.* Lammertink et al demonstrated that combining ultrasound and microbubbles could increase intracellular cisplatin and radiosensitivity in a head and neck cancer cell line.^14^ A recent in vitro experiment also showed that ultrasound-mediated drug delivery could be used to improve cellular gemcitabine uptake.^15^ Spatial localization of drug deposition can be achieved by loading drugs either into or onto the microbubble coating, using either chemical conjugation or physical methods, according to the drug properties.^10^ Application of ultrasound then causes local release of the drug from the microbubbles, followed by enhanced deposition. The ultrasound beam can be tightly focused onto the target volume, such that the microbubbles only interact with ultrasound in that volume. The feasibility of ultrasound-mediated gemcitabine delivery through coadministration has been shown in pancreatic cancer treatment both preclinically^13^ and clinically^16^^,^^17^ and for conjugation of gemcitabine onto microbubbles in murine pancreatic cancer.^18^

In this study, we investigated both gemcitabine delivery approaches in combination with radiation therapy using the Small Animal Radiation Research Platform (SARRP, from Xstrahl Ltd). We measured tumor growth delay and both systemic and acute normal-tissue toxicity in an orthotopic MIBC model. The results were compared with conventional gemcitabine-based chemoradiation therapy to determine whether these approaches could also maintain tumor growth delay while reducing toxicity.

## Methods and Materials

All animal work was carried out in accordance with UK Home Office Guidelines, per the Animal Research: Reporting of In Vivo Experiments guideline 2.0,^19^ and approved by the University of Oxford’s Animal Welfare and Ethical Review Body under project licenses P4B738A3B and P8484EDAE. All mice were purchased from Charles River Laboratories UK Ltd (Bristol, UK).

### Cell culture and chemicals

The human bladder cancer cell line, RT112, was obtained from the German Collection of Microorganisms and Cell Cultures GmbH in 2017 and cultured in RPMI-1640 medium supplemented with 10% (v/v) fetal bovine serum and 1% penicillin/streptavidin (Gibco, Life Technologies Ltd, Renfrew, UK). Cells were regularly tested for mycoplasma and found to be negative. 1,2-dipalmitoyl-sn-glycero-3-phosphocholine (DPPC), 1,2-distearoyl-sn-glycero-3-phosphoethanolamine-N-[biotinyl(polyethylene glycol)-2000] (DSPE-PEG(2000)Biotin), 1,2-distearoyl-sn-glycero-3-phosphocholine (DSPC), and 1,2-distearoyl-sn-glycero-3-phosphoethanolamine-N-[carboxy(polyethylene glycol)-2000] (DSPE-PEG(2000)) were obtained from Avanti Polar Lipids, Inc (Alabaster, AL). Gemcitabine hydrochloride (50 mg, Y0000657), cholesterol (>99% from egg white, C8667, Chol), and chloroform were purchased from Sigma-Aldrich (Dorset, UK). DPPC, DSPE-PEG(2000)Biotin, DSPC, and cholesterol were dissolved in chloroform and stored at –20^o^C before use.

### Synthesis of biotinylated gemcitabine

Because gemcitabine is hydrophilic, conjugation of gemcitabine onto microbubbles required structural modification. Chemical modification of gemcitabine is commonly used to improve the pharmacokinetic parameters, and several prodrugs have been used in clinical trials.^20^ Their design generally focuses on improving the rapid deamination of the amine group (N-4 position) or overcoming the drug resistance.^21^ In our approach, gemcitabine was biotinylated on the 5' position of the deoxyribose group to enable its bioconjugation onto microbubbles through avidin-biotin linkage.^18^ The biotin group can be cleaved by tissue or blood esterases, and this converts the biotinylated gemcitabine to gemcitabine.

Biotinylated gemcitabine was synthesized by sequential tert-butyloxycarbonyl (BOC) protection of the 3'-hydroxyl group (48 hours at room temperature in dioxane/water 5:1) and the cytosine NH (70 hours at 40^o^C in dioxane) of gemcitabine, followed by Steglich esterification of the 5'-hydroxyl group with biotin (24 hours at room temperature in dichloromethane) and deprotection with trifluoroacetic acid in dichloromethane.

### Microbubble preparation

Microbubbles were prepared by mixing DSPC, DSPE-PEG(2000), and DSPE-PEG(2000)Biotin at a molar ratio of 82:9:9 (all chemicals dissolved in chloroform). Chemicals were mixed in a glass vial and dried overnight on a hot plate at 50^o^C. The films were then resuspended with phosphate-buffered saline (PBS) at the lipid concentration of 20 mg/mL at 100^o^C. The suspension was sonicated at room temperature for 120 seconds at 20% amplitude using a 20 kHz ultrasonic cell disruptor (Probe sonicator, Q125 Sonicator, Qsonica, Newtown, CT). A second sonication was conducted for 30 seconds at 60% amplitude under perfluorobutane (F2 Chemicals Ltd, Preston, UK), resulting in the production of microbubbles. The vials were then placed on ice for 3 minutes. Avidin (CAT# 189725 from EMD Millipore) was first dissolved in PBS and then mixed with the microbubbles by gently inverting the vials several times (final avidin concentration = 1 mg/mL). The suspension was centrifuged at 280⋅g for 5 min at 4^o^C. After centrifugation, the unbound avidin in the solution was separated from the floating microbubbles and the microbubbles were replenished with an equal volume of PBS. The diameters and concentration of microbubbles were measured by light microscopy and a custom MATLAB (Mathworks Inc, Natick, MA) script, as described by Sennoga et al.^22^ Microbubbles were then mixed with gemcitabine or biotinylated gemcitabine dissolved in PBS.

### Cell preparation and orthotopic tumor induction

The MIBC model was generated by orthotopically injecting CD1-nude mice (6-8 weeks old, female) with human RT112 bladder cancer cells under ultrasound guidance, as described by Jäger et al.^23^ Briefly, RT112 cells were prepared in phenol red-free Matrigel (354262, High Concentration [HC] Phenol-Red Free LDEV-Free, Corning Ltd, Deeside, UK) and PBS 1:2, and 50 μL (concentration = 1.5x10^7^ cells/mL) was injected into the bladder wall.

At 5 to 7 days after tumor inoculation, the mean tumor volume was 45.96 ± 1.93 mm^3^ measured over 43 mice for the tumor-growth delay experiment (averaged weight per animal: 25.38 ± 0.30 g). Mice were then grouped by random selection, and members of each group were subjected to 1 of the following treatments: (1) sham control; (2) ultrasound and microbubbles only (MB + US); (3) radiation only (IR); (4) ultrasound, microbubbles, and radiation (MB + US + IR); (5) gemcitabine intravenous injection (10 mg/kg) and radiation (gem + IR); (6) ultrasound with coadministration of gemcitabine (10 mg/kg) and microbubbles and radiation (gem + MB + US + IR); or (7) ultrasound with gemcitabine-conjugated microbubbles and radiation (gembioMB + US + IR, with 10 mg/kg gemcitabine and microbubble concentration = 3x10^8^ bubbles/mL). The ultrasound and radiation exposure protocols are described below. Tumor volume was measured using 3D B-mode imaging (40 MHz transmit frequency, 100% power, 22 dB acquisition gain) from a Vevo 3100 Preclinical Imaging System with a MX550D probe (FUJIFILM VisualSonics, Toronto, ON) at least twice per week. Mice were weighed daily for the first 4 days, then 3 times a week for the remaining experimental period. Mice were culled when the tumor reached 300 mm^3^, when they developed hematuria, or 53 days posttreatment, whichever occurred earliest.

For the normal-tissue toxicity experiment, 5 to 7 days after tumor inoculation, mice were grouped by random selection, and members of each group underwent 1 of the following treatments: (1) sham control; (2) IR; (3) MB + US + IR (microbubble concentration = 3 × 10^8^ bubbles/mL); (4) gem + IR (10 mg/kg gemcitabine); (5) gem + MB + US + IR (10 mg/kg gemcitabine and microbubble concentration = 3 × 10^8^ bubbles/mL); (6) gemcitabine-conjugated microbubbles and radiation (gembioMB + IR, with 10 mg/kg gemcitabine and microbubble concentration = 3x10^8^ bubbles/mL); or (7) gembioMB + US + IR, with 10 mg/kg gemcitabine and microbubble concentration = 3x10^8^ bubbles/mL. The ultrasound and radiation exposure protocols are described below. Mice were culled 3.75 days posttreatment and subjected to the modified crypt assay as described below. The concentration of the gemcitabine was verified by a high performance liquid chromatography.

### Ultrasound exposure

**Fig. 1:**
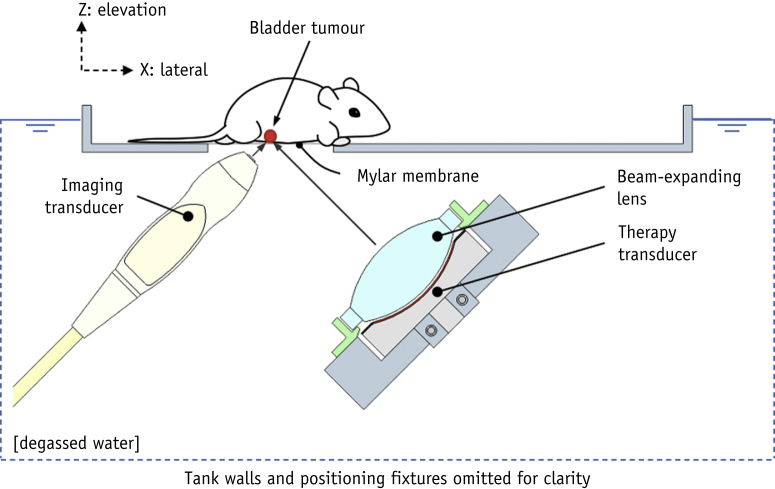
Schematic of the image-guided ultrasound treatment system.

An image-guided ultrasound treatment system was developed to locate the bladder tumor and deliver the drug (Fig. 1). In this system, an 8 MHz imaging transducer (L11.5v, Verasonics, Kirkland, WA) was used to locate the bladder tumor and a 1.1 MHz annular focused therapeutic transducer (H102, bandwidth = ±220 kHz, aperture = 64 mm, geometric focal distance = 63.2 mm, Sonic Concepts, Bothell, WA) was used to deliver the therapeutic ultrasound beam. The beam width (full width at half-maximum) of this transducer in the lateral and elevation planes were measured to be 1.2 mm each. The therapeutic transducer was connected to a radio frequency power amplifier (1040L, E&I Ltd., Rochester, NY), which was driven by an arbitrary waveform generator (33220A, Agilent, Santa Clara, CA) with a 1.1 MHz sine wave. The imaging transducer was placed at right angles to the therapeutic transducer, and both transducers were positioned at 45^o^ to the horizontal to ensure their beams intersected at the tumor site. The therapeutic transducer was aligned to the targeted area using a 0.2-mm-diameter needle hydrophone (Onda HN0200, Onda Corporation, Sunnyvale, CA). The imaging transducer was operated by the Vantage system (Verasonics, Kirkland, WA). The beam width of the therapeutic transducer was expanded with a biconvex lens to enable coverage of the whole bladder. With the expansion lens, the beam width was measured to be 6 mm in elevation and lateral planes, and the focal distance was moved to 110 mm. The propagation distance between the target site and the therapeutic transducer was 110 mm, and the beam width was 6 mm in elevation and lateral planes. The position of the therapy transducer was fixed. Before the start of the experiments, the imaging transducer was aligned to the focal region of the therapy transducer using a needle hydrophone (Onda HN0200). The position of the tip of the needle hydrophone was marked in the ultrasound image, and the target site was aligned to the same position using image guidance. The therapeutic ultrasound exposure parameters were as follows: 1.1 MHz center frequency, 1 MPa peak negative pressure, 1% duty cycle, and 0.5 Hz pulse repetition frequency. The acoustic settings were chosen to allow for microbubble reperfusion after microbubble destruction. Microbubble-drug mixtures were injected via a syringe pump (flow rate for each injection = 0.5 mL/min, AL-1000, World Precision Instruments) 8 times, using 25 μL of suspension each time, and 40 therapeutic ultrasound pulses were applied between each injection to burst the microbubbles. Two hundred microliters of suspension were injected to each mouse. The Vantage research platform (Verasonics, Kirkland, WA) was used to control the function generator for the therapeutic pulse delivery as well as the syringe pump for the microbubble injection to guarantee routine and accurate administration of both.

### Radiation with the Small Animal Radiation Research Platform

Approximately 6 hours after gemcitabine and/or ultrasound treatment, mice were irradiated using the SARRP (Xstrahl Ltd), delivering 220 kV x-rays, 13 mA, half-value layer 0.794 mm Cu. For tumor growth delay experiments, mice were irradiated vertically as described by Groselj et al^24^ with temperature maintained homeothermically,^25^ to a dose of 6 Gy, using a 356° arc treatment and with a circular collimator, typically between 5.2 and 8.4 mm, to cover the whole bladder and minimize any normal-tissue damage. For the acute normal-tissue toxicity experiments, to cover a greater volume of bowel, mice were treated supine, and 12 Gy was delivered to the lower abdomen including the small intestine by the SARRP, using a 178° arc treatment and with a 14-mm circular collimator.

### Modified crypt assay

The “Swiss roll” crypt assay was conducted as described by Groselj et al.^24^ The small intestines from the mice were collected and rolled up to provide “Swiss rolls” for histology. Samples were cut into 5-μm slides and subjected to hematoxylin and eosin staining. The slides were scanned using an Aperio CS2 digital pathology slide scanner (Leica Biosystems, Wetzlar, Germany) and analyzed anonymously using Aperio ImageScope software (Leica Biosystems). The percentage of crypts surviving was calculated as follows:

$$\frac{Number\;of\;crypts\;from\;irradiated\;mice}{Number\;of\;crypts\;from\;control\;mice}\times 100$$

### Statistical analysis

Statistical analyses were performed with Prism (GraphPad), and results were presented as mean ± SEM. Multiple group comparisons were made using 1-way ANOVA followed by a post hoc Tukey multiple comparison test. The Kaplan-Meier curve was used to present the time to triple tumor volume. One mouse from the gem + MB + US + IR group and 1 from the gembioMB + US + IR group survived to the endpoint (53 days posttreatment) without reaching the 3-fold volume increase. Two mice from the gembioMB + US + IR group were censored (culled) owing to hematuria, 1 at day 33 and 1 at day 45, but the tumors had not reached a 3-fold volume increase. Those times were also included as the times to triple tumor volume. The log-rank test was conducted on the Kaplan-Meier curve before day 32 to determine statistical significance (*P* < .05). Pearson correlation was used to study the relationship between 2 parameters with a 2-tailed *P* value.

## Results

### Combining ultrasound-mediated drug delivery and radiation demonstrated improved tumor growth delay similar to conventional gemcitabine-based chemoradiation therapy

**Fig. 2:**
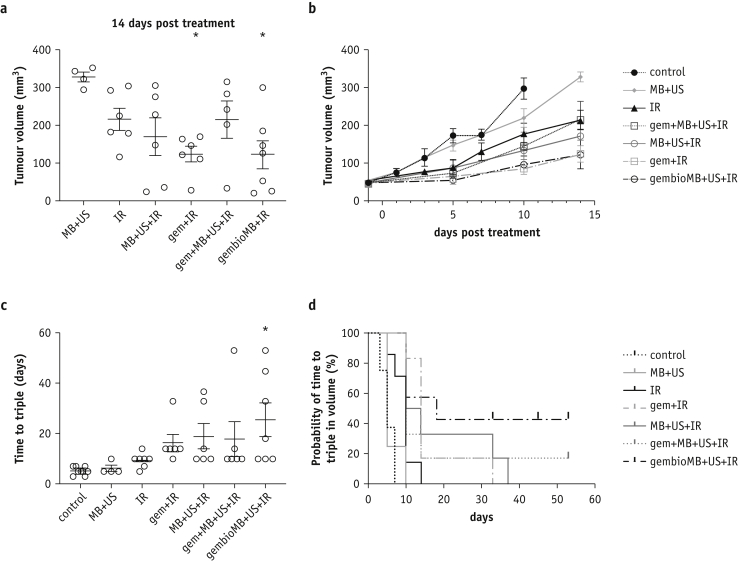
Orthotopic muscle-invasive bladder tumor growth delay observed by combining ultrasound-mediated drug delivery and radiation. (a) Tumors from mice treated with gem + IR and gembioMB + US + IR were still significantly smaller than those in mice treated with MB + US 14 days posttreatment; ∗*P* < .05 compared with control. (b) Tumor growth curve until day 14 posttreatment. (c) Mice treated with gembioMB + US + IR had prolonged time to triple tumor volume compared with the control group; ∗*P* < .05 compared with control. (d) Kaplan-Meier survival curve showing plots of time to triple tumor volume after the treatments; control versus IR: *P* < .01; IR versus gem + IR and IR versus gembioMB + US + IR: *P* < .05. n = 8 for control; n = 4 for MB + US; n = 6 for IR, gem + IR, MB + US + IR, and gem + MB + US + UR; and n = 7 for gembioMB + US + IR. *Abbreviations:* gem = gemcitabine; gembioMB = gemcitabine-conjugated microbubbles; IR = radiation; MB = microbubbles; US = ultrasound.

**Fig. E1:**
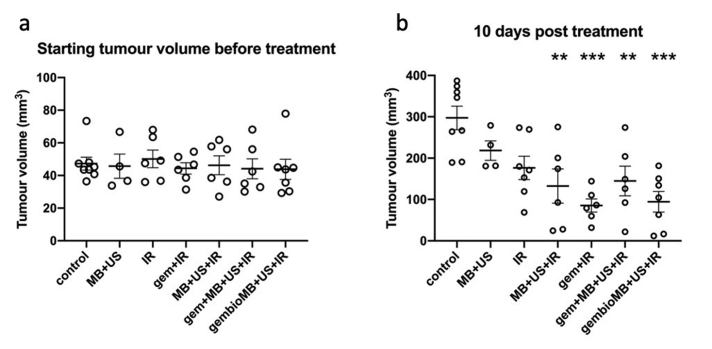


The tumor size was approximately 30 to 80 mm^3^ before the treatment started and showed no difference among different treatment groups (Fig. E1a). Ten days posttreatment, more than half of the mice from the control group had reached the humane endpoint (tumor volume of 350 mm^3^). Compared with the controls showing a mean tumor size of 297.3 ± 28.4 mm^3^, significant tumor growth delay was found in the following groups: gem + IR (85.34 ± 16.0 mm^3^, *P* < .001), MB + US + IR (132.5 ± 41.2.0 mm^3^, *P* < .01), gem + MB + US + IR (144.7 ± 35.8 mm^3^, *P* < .01), and gembioMB + US + IR (94.6 ± 25.0 mm^3^, *P* < .001) (Fig. E1b). No difference in tumor volume was found between the control mice and mice subjected to radiation only (IR, 176.5 ± 28.3 mm^3^) or to microbubbles and ultrasound without drug or irradiation (MB + US, 218.4 ± 23.5 mm^3^) (Fig. E1b). At 14 days posttreatment, compared with those in the MB + US only group (mean tumor volume, 328.3 ± 12.9 mm^3^), only mice subjected to conventional chemoradiation therapy (gem + IR, 123.9 ± 21.3 mm^3^) or ultrasound with gemcitabine conjugated microbubbles and irradiation (gembioMB + US + IR, 122.3 ± 37.9 mm^3^) still showed significant tumor growth delay (Fig. 2a and 2b, *P* < .05 for each). Overall, the time to triple tumor volume for gembioMB + US + IR groups (mean = 25.6 ± 6.8 days; 95% CI = 8.8-42.3 days) was also significantly prolonged compared with the control group (mean = 5.25 ± 0.6 days; 95% CI = 3.9-6.6 days) (Fig. 2c; control versus gembioMB + US + IR, *P* < .05).

The Kaplan-Meier curve for time to triple volume showed significantly delayed tumor growth in the gem + IR and gembioMB + US + IR groups compared with the IR-only group (Fig. 2d, *P* < .05 for each). However, no significant difference in the time to triple volume was detected between the gem + IR group and all 3 ultrasound-mediated drug delivery groups (MB + US + IR, gem + MB + US + IR, and gembioMB + US + IR) (Fig. 2d). Whereas most of the mice in the IR-only group had their tumor volume triple by 10 days posttreatment, the median time to triple volume for mice subjected to gem + IR and gembioMB + US + IR was 14 and 18 days, respectively (*P* < .05 for each). The median time to triple volume for MB + US + IR was 12 days but was only marginally different from the IR-only group (*P* = .05). Similar to the IR-only group, the gem + MB + US + IR group also showed a median of 10 days.

### Combining ultrasound-mediated drug delivery and radiation demonstrated transient weight loss and was correlated with tumor growth delay

**Fig. 3:**
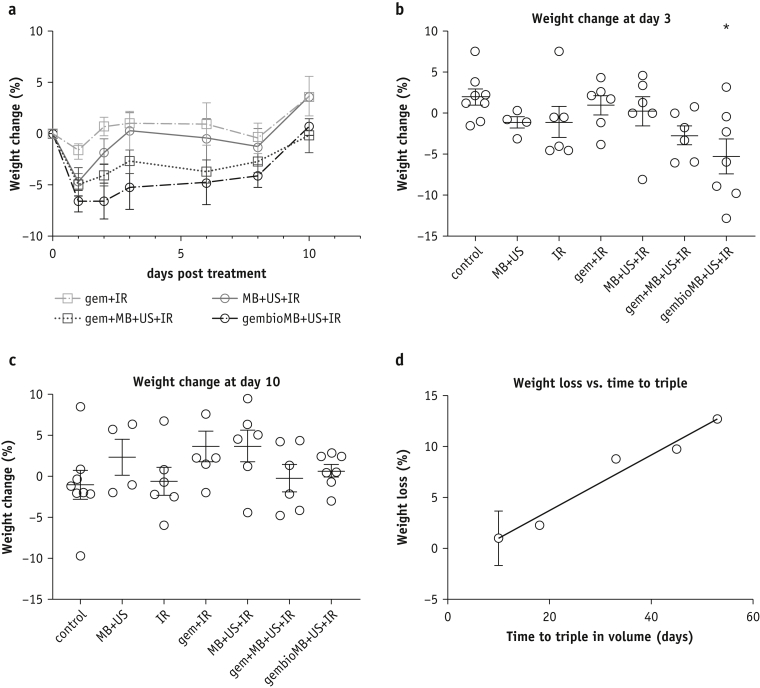
Weight change in mice bearing orthotopic muscle-invasive bladder tumor posttreatment. (a) Daily weight change after treatment for conventional chemoradiation therapy (gem + IR) and IR with the 3 ultrasound-mediated drug delivery methods (MB + US + IR, gem + MB + US + IR, and gembioMB + US + IR). (b) Three days posttreatment, only mice from the gembioMB + US + IR group showed significant weight loss compared with control; ∗*P* < .05 compared with control. (c) All weight loss was resolved 10 days posttreatment. (d) A positive correlation (*P* < .05; R^2^ = 0.76) was found between weight loss at day 3 and time to triple volume in the gembioMB + US + IR group. n = 8 for control; n = 4 for MB + US; n = 6 for IR, gem + IR, MB + US + IR, and gem + MB + US + UR; and n = 7 for gembioMB + US + IR. *Abbreviations:* gem = gemcitabine; gembioMB = gemcitabine-conjugated microbubbles; IR = radiation; MB = microbubbles; US = ultrasound.

Animal weight was measured as an indicator of systemic treatment toxicity. Weight was presented as the percentage difference in weight before and each day after treatment (Fig. E2a). Weight loss was observed from all the treatment groups 1 day after treatment (Fig. 3a). Mice from the gem + IR group experienced a weight loss of only 1.8 ± 0.7% on day 1, but this was not significantly different from the control. One day after treatment, compared with the control, we found a significant weight loss for the 3 ultrasound-mediated drug delivery groups: MB + US + IR (4.7 ± 1.4%, *P* < .05), gem + MB + US + IR (5.0 ± 1.1%, *P* < .05), and gembioMB + US + IR (6.6 ± 1.0%, *P* < .001) (Fig. E2b). Significantly different weight loss (5.2 ± 2.1%) for the gembioMB + US + IR group compared with the control group lasted until day 3 (Fig. 3b; control versus gembioMB + US + IR, *P* < .05). By day 10, most mice from all groups had recovered their weight to within 5% of baseline (Fig. 3c). However, we found a positive correlation, as more weight loss in day 3 was associated with longer time to triple tumor volume (Fig. 3d; *P* < .05; R^2^ = 0.76).

### Ultrasound-mediated gemcitabine delivery reduces acute normal-tissue toxicity from chemoradiation therapy

**Fig. 4:**
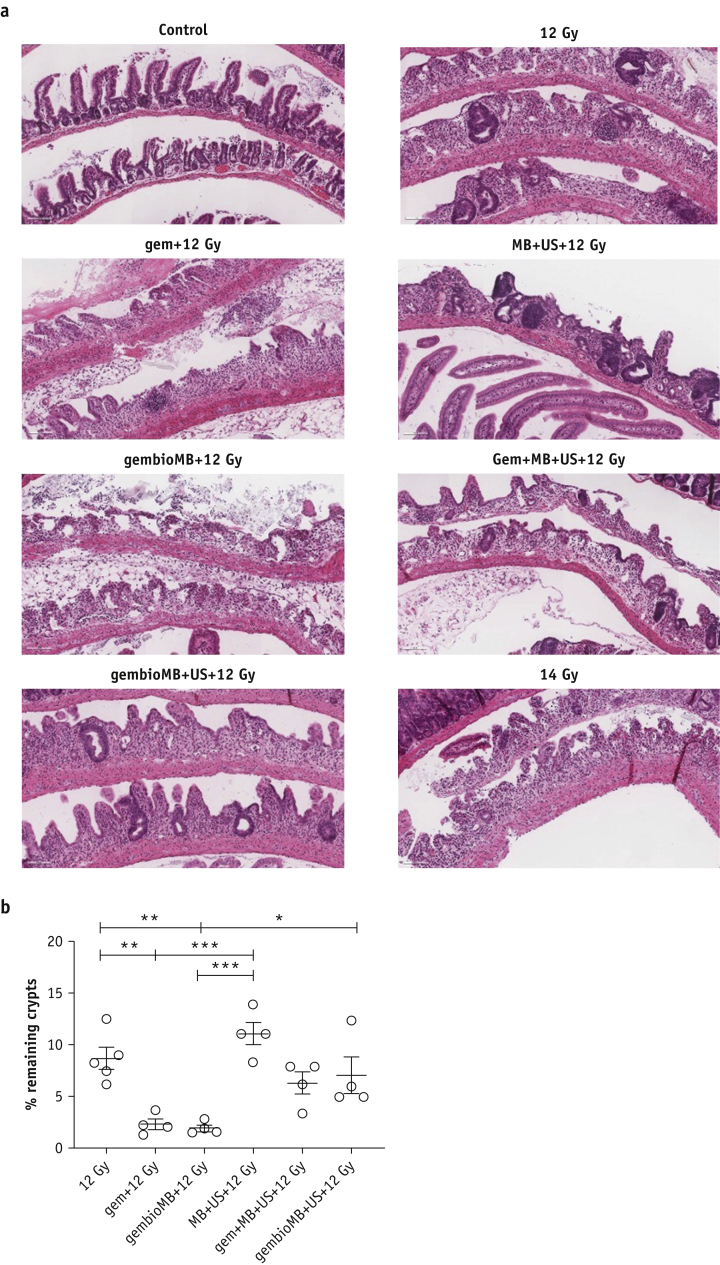
Acute normal tissue toxicity. (a) Representative images of intestinal crypt from each treatment group. Scale bar represents 100 μm. (b) Assessment of acute normal tissue toxicity using crypt survival. Conventional chemoradiation therapy (gem + 12 Gy) or gembioMB + 12 Gy resulted in significant reduction in crypt survival compared with IR at 12 Gy only. There was no difference between 12 Gy and the 3 ultrasound-mediated drug delivery methods (MB + US + 12 Gy, gem + MB + US + 12 Gy, and gembioMB + US + 12 Gy), indicating that exacerbation of normal-tissue toxicity by addition of chemotherapy could be avoided; ∗*P* < .05, ∗∗*P* < .01, ∗∗∗*P* < .001. n = 5 for 12 Gy, and n = 4 for all of the other groups. *Abbreviations:* gem = gemcitabine; gembioMB = gemcitabine-conjugated microbubbles; IR = radiation; MB = microbubbles; US = ultrasound.

We then evaluated acute normal-tissue toxicity by using the SARRP to cover a greater volume of bowel. Mice were irradiated in a supine position, and the beams were positioned to cover only part of the bladder and to include the surrounding bowel tissue. We used a 12 Gy radiation dose, as 6 Gy was insufficient to induce discernible damage in the intestine. Mice were culled 3.75 days posttreatment, and their small intestines were collected to make Swiss rolls for histology. The most damaged areas of the intestine in each group were identified, and the crypts from those areas were counted. Compared with mice irradiated with 12 Gy, which had 8.7 ± 1.1% remaining crypts, significant crypt loss was found in the conventional gem + IR group and the gembioMB + IR group, with only 2.3 ± 0.5% and 1.9 ± 0.3% crypts remaining, respectively (Fig. 4b; 12 Gy versus gem + 12 Gy or gembioMB + 12 Gy, *P* < .01). The crypt damage in these groups was similar to the damage caused by 14 Gy irradiation (Fig. 4a). On the other hand, no significant change in crypt survival was found between IR only and all 3 ultrasound-mediated drug delivery approaches (Fig. 4b). The MB + US + 12 Gy group had 11.0 ±1.1% remaining crypts, the gem + MB + US + 12 Gy group had 6.3 ± 1.1% remaining crypts, and the gembioMB + US + 12 Gy group had 7.0 ± 1.8% remaining crypts. Significant differences were found between gem + IR or gembioMB + IR and MB + US + IR (Fig. 4b; *P* < .001 for each). A marginal difference was detected between gem + IR and gembioMB + US + IR (*P* = .06), and a significant difference was detected between mice treated with gembioMB + IR and gembioMB + US + IR (Fig. 4b; *P* < .05), suggesting that ultrasound is indeed essential in reducing normal-tissue toxicity.

## Discussion

In this study, we tested the hypothesis that using microbubbles and ultrasound reduces local toxicity compared with conventional chemoradiation without compromising treatment efficacy. The conventional chemoradiation was modeled by intravenous injection of 10 mg/kg gemcitabine 6 hours before irradiation (gem + IR). Under the same systemically administrated gemcitabine dose and radiation regimen, we explored 2 different gemcitabine administration approaches to improve the gemcitabine delivery to our in vivo model using ultrasound-mediated drug delivery: coadministration of microbubbles and gemcitabine and conjugation of gemcitabine onto microbubbles via avidin-biotin linkage. As described in the introduction, both methods have been previously shown to be feasible in treating pancreatic cancer. We showed that both treatments were associated with tumor growth delay comparable with that of conventional chemoradiation. Although a transient systemic toxicity, as indicated by the weight loss, was found in both groups, there was also reduced acute intestinal-tissue toxicity from the conventional chemoradiation.

The advantage of combining ultrasound-mediated drug delivery using coadministration of microbubbles and gemcitabine with ionizing radiation (gem + MB + US + IR) was its straightforward implementation in clinical practice. In our study, this technique initially showed comparable tumor growth delay compared with the gem + IR groups (Fig. E1a), but the effect was only comparable with that of IR at a later time point (Fig. 2). Weight loss compared with the control group was significant 1 day after treatment but not significant after day 2. This method also reduced the intestinal toxicity compared with the gem + IR group (Fig. 4). On the other hand, we showed that gemcitabine-conjugated microbubbles and ultrasound with radiation therapy (gembioMB + US + IR) is more efficient in delaying tumor growth. This technique showed tumor growth delay comparable with the gem + IR group, even at later time points (Fig. E1 and Fig. E2). Significant weight loss from this group was still found on day 3 but not after day 6 (Fig. 3). This approach showed reduced intestinal toxicity compared with that observed in the gem + IR group (Fig. 4). In addition, this approach improved crypt survival, compared with administering only gemcitabine-conjugated microbubbles (Fig. 4). The lack of ultrasound-mediated destruction of gemcitabine-conjugated microbubbles may result in exposure of nontumor tissues to gemcitabine for longer, potentially increasing uptake of the drug by those normal tissues.

We also tested the direct effects of ultrasound-mediated drug delivery by applying microbubbles and ultrasound without gemcitabine. Previous studies showed that this application could also radiosensitize tumors, potentially through effects of vascular damage, when the treatment was delivered simultaneously with or no more than 6 hours before radiation therapy.^26^, ^27^, ^28^, ^29^ Applying ultrasound-mediated delivery without radiation did not achieve improved delay in tumor growth (Fig. 2). We found that when combined with radiation, this approach could reduce intestinal tissue toxicity (Fig. 4), achieving a delay in tumor growth and systemic toxicity similar to that observed using gem + MB + US + IR (Fig. E1 and Fig. 2).

We showed that combining ultrasound-mediated drug delivery with radiation (MB + US + IR, gem + MB + US + IR, or gembioMB + US + IR), although it resulted in transient systemic toxicity, was safe. Only transient weight loss, which resolved at approximately10 days posttreatment, was found during the study (Fig. 3). Previous reports have shown that ultrasound can cause gut damage, such as reduction of mitotic cells and petechiae.^30^^,^^31^ It is possible that the transient weight loss observed in this study was a result of mechanical damage on the gut by ultrasound, but this did not significantly enhance the acute intestinal toxicity when combined with radiation. We also found the treatment-associated weight loss was positively correlated with tumor growth delay for the gembioMB + US + IR group, as more weight loss was associated with longer tumor growth delay. Some studies have shown that ultrasound may have immunomodulatory effects.^32^, ^33^, ^34^ As CD1-nude mice still have innate immunity, it is possible the that treatment may cause cytokine expression from the local treatment area to the circulation, modulating other physiological effects and causing weight loss.^35^ Further studies are needed to understand whether the local effect of tumor growth delay and the systemic effect of weight loss can be attributed to immune response from the treatment.

Clinically, normal-tissue toxicity limits the ability to cure cancers by limiting the dose of chemotherapeutic agents or radiation that can be delivered. To improve the efficacy of chemoradiation therapy, the 2 commonly used approaches are finding alternative radiosensitizers with less normal-tissue toxicity or using radioprotectors. The former approach generally requires development of new drugs, which is time and resource consuming.^36^ Radioprotectors are usually administered before radiation exposure. Several radioprotectors have been developed to reduce acute intestinal radiation injury, but most are still in the preclinical phase of development and have not been tested in combination with chemoradiation.^37^ This study provides an alternative approach by combining ultrasound-mediated drug delivery with chemoradiation therapy. We showed the efficacy in an orthotopic model of muscle-invasive bladder cancer, which is more clinically relevant than the models used in most chemoradiation preclinical studies, namely ectopic xenograft tumor models, coupled with normal-tissue toxicity studies in the relevant organs that display dose-limiting toxicity. We showed that we were able to reduce normal-tissue toxicity without compromising tumor control, using a common and clinically approved chemoradiation agent, gemcitabine. The technology can be immediately applied to other clinically available chemoradiation agents to improve the efficacy of chemoradiation therapy.

## Conclusion

Using microbubbles and ultrasound to deliver chemoradiation therapy was found to reduce local normal-tissue toxicity compared with conventional chemoradiation without compromising treatment efficacy. Conjugating gemcitabine onto microbubbles was found to be more effective than coadministering microbubbles and gemcitabine in terms of long-term delay in tumor growth. Ultrasound and microbubbles alone were found to produce a similar delay in tumor growth but with reduced toxicity compared with coadministered microbubbles and gemcitabine. Combing ultrasound-mediated drug delivery with radiation resulted in transient systemic toxicity, but this resolved 10 days posttreatment. As ultrasound and microbubbles have been extensively used in the clinic for both diagnosis and therapeutic purposes, clinical trials to further assess the safety and efficacy of incorporating ultrasound-mediated drug delivery into chemoradiation therapy should be considered.

## Figures and Tables

**Fig. E2:**